# Data and Theory Point to Mainly Additive Genetic Variance for Complex Traits

**DOI:** 10.1371/journal.pgen.1000008

**Published:** 2008-02-29

**Authors:** William G. Hill, Michael E. Goddard, Peter M. Visscher

**Affiliations:** 1 Institute of Evolutionary Biology, School of Biological Sciences, University of Edinburgh, Edinburgh, United Kingdom; 2 Faculty of Land and Food Resources, University of Melbourne, Victoria, Australia; 3 Department of Primary Industries, Victoria, Australia; 4 Queensland Institute of Medical Research, Brisbane, Australia; North Carolina State University, United States of America

## Abstract

The relative proportion of additive and non-additive variation for complex traits is important in evolutionary biology, medicine, and agriculture. We address a long-standing controversy and paradox about the contribution of non-additive genetic variation, namely that knowledge about biological pathways and gene networks imply that epistasis is important. Yet empirical data across a range of traits and species imply that most genetic variance is additive. We evaluate the evidence from empirical studies of genetic variance components and find that additive variance typically accounts for over half, and often close to 100%, of the total genetic variance. We present new theoretical results, based upon the distribution of allele frequencies under neutral and other population genetic models, that show why this is the case even if there are non-additive effects at the level of gene action. We conclude that interactions at the level of genes are not likely to generate much interaction at the level of variance.

## Author Summary

Genetic variation in quantitative or complex traits can be partitioned into many components due to additive, dominance, and interaction effects of genes. The most important is the additive genetic variance because it determines most of the correlation of relatives and the opportunities for genetic change by natural or artificial selection. From reviews of the literature and presentation of a summary analysis of human twin data, we show that a high proportion, typically over half, of the total genetic variance is additive. This is surprising as there are many potential interactions of gene effects within and between loci, some revealed in recent QTL analyses. We demonstrate that under the standard model of neutral mutation, which leads to a U-shaped distribution of gene frequencies with most near 0 or 1, a high proportion of additive variance would be expected regardless of the amount of dominance or epistasis at the individual loci. We also show that the model is compatible with observations in populations undergoing selection and results of QTL analyses on F2 populations.

## Introduction

Complex phenotypes, including quantitative traits and common diseases, are controlled by many genes and by environmental factors. How do these genes combine to determine the phenotype of an individual? The simplest model is to assume that genes act additively with each other both within and between loci, but of course they may interact to show dominance or epistasis, respectively. A long standing controversy has existed concerning the importance of these non-additive effects, involving both Fisher [1] and Wright [2]. Estimates of genetic variance components within populations have indicated that most of the variance is additive [3],[4]. Increasing knowledge about biological pathways and gene networks implies, however, that gene-gene interactions (epistasis) are important, and some have argued recently that much genetic variance in populations is due to such interactions [5],[6],[7],[8]. It is important to distinguish between the observations of dominance or epistasis at the level of gene action at individual loci, exemplified by a table of genotypic values, and the observations of variance due to these components in analysis of data from a population. For example, at a completely dominant locus almost all the variance contributed is additive if the recessive gene is at high frequency [3],[4].

An understanding of the nature of complex trait variation is important in evolutionary biology, medicine and agriculture and has gained new relevance with the ability to map genes for complex traits, as demonstrated by the recent burst of papers that report genome-wide association studies between complex traits and thousands of single nucleotide polymorphisms (SNPs) [9],[10],[11],[12],[13]. Here we attempt to resolve the alternative sources of evidence on the importance of non-additive genetic variation. We evaluate the evidence from empirical studies of genetic variance components and indeed find that additive variance typically accounts for over half and often close to 100% of the total genetic variance. We then present new theory and results that show why this is the case even if there are non-additive effects at the level of gene action.

### Empirical Evidence for Additive and Non-Additive Genetic Variance

#### Estimation of Genetic Variance

The genetic variance *V*_G_ can be partitioned into additive (*V*_A_), dominance (*V*_D_), and a combined epistatic component (*V*_I_), which itself can be partitioned into two locus (*V*_AA_, *V*_AD_, and *V*_DD_) and multiple locus components (*V*_AAA_, etc.) [3],[4],[14],[15],[16],[17]. Estimation of additive and non-additive variance components utilises the observed phenotypic similarity of relatives and the expected contribution of additive and non-additive effects to that similarity [3],[4]. In addition to resemblance due to additive or non-additive genetic factors, relatives may resemble each other due to common environmental effects.

In an extremely large data set with very many different kinds of relationships present, it is possible in principle to partition variation into many components using modern statistical methods such as residual maximum likelihood [18] (REML) with the animal model [4],[19],[20]. In practice it is never possible to estimate many variance components with useful precision, however, not least because there is a high degree of confounding: for example, full sibs have a higher covariance for all single and multi-locus genetic components than do half sibs. The coefficients of epistatic components are small (e.g., *V*_AA_/16 for half-sibs), so estimates have high sampling error and there is little power to distinguish *V*_A_ from, say, *V*_AA_. Selection, assortative mating, and non-genetic covariances also confound estimates. Consequently, there are few accurate estimates of non-additive variance components but there is indirect evidence. For instance, a narrow sense heritability value (*h* ^2^ = *V*_A_/*V*_P_) of one-half, typical for many traits, implies that dominance, all the vast number of epistatic components, and the environmental component, collectively contribute no more than *V*_A_. Similarly if the heritability is only a little less than the repeatability (the phenotypic correlation of repeated measures), all non-additive genetic variances and the permanent environmental variance together comprise this small difference. With these caveats we summarise data of various types.

#### Laboratory Animals and Livestock

The extensive data on experimental organisms show a range of heritability, higher for morphological than fitness associated traits, averaging as follows [21]: morphology - 0.46, physiology (e.g., oxygen consumption, resistance to heat stress) - 0.33, behaviour - 0.30, and life history - 0.26.

There have been extensive estimates of heritability for traits of livestock. For example, for beef cattle, these averaged: post-weaning weight gain 0.31, market weight for age 0.41, backfat thickness 0.44 [22]. In general for morphological traits, such as carcass fatness, egg weight in poultry or fat and protein content of cow's milk, a heritability of 0.5 or so is the norm, whereas for growth traits or milk yield 0.25–0.35 is more typical [23]. These estimates of heritability from half-sib correlations could be biased upwards by additive epistatic terms, but they can not account for estimates of heritability over 25%. Furthermore, estimates of realised heritability from response to selection [3] are not biased in that way, because epistatic components do not contribute to long term selection response [24], and estimates of realised heritability range up to 0.5 for fat content of mice, for example [25].

There are a number of cases where it can be shown directly that *V*_A_ contributes almost all of *V*_G_ and indeed almost all of *V*_P_. For bristle number in *Drosophila melanogaster*, the phenotypic correlation between abdominal segments, which, assuming they are influenced by the same genes, estimates *V*_G_/*V*_P_, is only a little higher than the heritability, indicating that *V*_A_/*V*_G_∼0.8 [26]. For finger ridge count (in humans), estimates of heritability are close to one and consistent from different sorts of relatives [27]. Even for lowly heritable traits such as litter size in pigs, the repeatability is little higher than the heritability, implying that most genetic variance is additive [28]. Whilst there is a clear relationship between heritability and type of trait, it should be noted that low heritability does not imply low genetic variance: the evolvability (√*V*_A_/*mean*) is higher for fitness than morphological traits [29], and even for estimates of fitness itself or traits closely related to it, additive genetic variance is present [30],[31].

There are rarely good direct estimates of epistatic or dominance variance because these variance components are usually estimated from full-sibs and therefore confounded with the common environment shared by full sibs. However, if the heritability is high, the space for them is limited.

Experiments on inbreeding depression provide some evidence on the importance of non-additive effects. Inbreeding depression implies directional dominance in gene effects but, for a given rate of inbreeding depression, as the number of loci increases and the gene frequencies move toward 0 or 1.0, the dominance variance decreases towards zero. Consequently, the importance of inbreeding depression for traits related to fitness is not evidence that the dominance variance is large. The observed linearity of inbreeding depression with inbreeding co-efficient is easiest to explain with directional dominance but not with DD or higher order epistatic effects because these would cause non-linearity unless they happened to exactly cancel each other out.

#### Twin Studies in Humans

In contrast to studies of sibs and more distant relatives, identical twins can provide estimates of *V*_G_. The classical twin design of samples of monozygotic (MZ) and dizygotic (DZ) twin pairs has been used extensively to estimate variance components for a wide range of phenotypes in human populations. The primary statistics from these studies are the correlations between MZ pairs (*r*_MZ_) and between DZ pairs (*r*_DZ_). If twin resemblance due to common environmental factors is the same for MZ and DZ twins then *r*_MZ_>*r*_DZ_ implies that part of the resemblance is due to genetic factors and *r*_MZ_>2*r*_DZ_ implies the importance of non-additive genetic effects. Conversely, *r*_MZ_<2*r*_DZ_ implies that common environmental factors cause some of the observed twin resemblance. Sophisticated variance component partitioning methods to estimate components of additive, non-additive and common environmental effects are used widely [32], but all rely on the strong assumptions that resemblance due to common environmental effects is the same for MZ and DZ twins. Attempts to test this hypothesis have not found any evidence to reject it [33],[34]. Nevertheless, even accepting this assumption about common environmental variance, in the classical twin design there are only two primary statistics and three or more variance components cannot be estimated without making additional assumptions.

**Figure 1:**
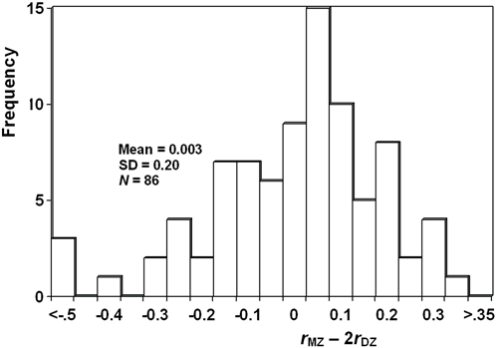
Distribution of *r*_MZ_−2*r*_DZ_ for all traits on human twins. Data are from published papers by N.G. Martin and colleagues of the Queensland Institute of Medical Research, Brisbane (www.genepi.edu.au). Across a wide variety of traits the mean difference between the monozygotic twin correlation and twice the dizygotic twin correlation is close to zero, which is consistent with predominantly additive genetic variance and the absence of a large component of variance due to common environmental effects.

We summarised the MZ and DZ correlations for a wide variety of phenotypes from published twin studies from a single productive laboratory in Australia (genepi.qimr.edu.au). The criteria were that each study must have more than 100 MZ and more than 100 DZ pairs and that the study subjects were Australian twins. For non-continuous traits, studies were included only if they reported polychoric or tetrachoric correlations. In total, 86 phenotypes qualified of which 42 were clinical measures of quantitative traits (including, for example, blood pressure, biochemical measures in blood, body-mass-index, height, tooth dimensions; a full list of phenotypes is available upon request). The MZ and DZ correlations are summarised in Table 1. The correlations were not separated according to the sex of the individuals in all studies; but for those that did separate the sexes, the overall MZ and DZ correlations were calculated as an average, weighted by the total number of pairs. The distribution of *r*_MZ_−2*r*_DZ_ across all 86 phenotypes is shown in Figure 1. On average the MZ correlation is about twice the DZ correlation across a wide range of phenotypes. If we consider only clinically measured phenotypes and ignore opposite-sex twins then the MZ correlation is clearly less than twice the DZ correlation (Table 1). It is possible but unlikely that the variance due to common environmental factors, assortative mating and non-additive genetic factors exactly cancel each other out by chance. Thus the simplest explanation of the results is that additive variance explains most of the observed similarity of twins and non-additive variance is generally of small magnitude and cannot explain a large proportion of the genetic variance.

**Table 1:** Meta-analysis of MZ and DZ correlations in humans^a^.

Group	All phenotypes		Clinically measured phenotypes	
	No. traits	*r*	No. traits	*r*
MZ females	58	0.61	24	0.76
MZ males	48	0.65	24	0.75
DZ females	58	0.34	24	0.45
DZ males	48	0.36	24	0.43
OS pairs^b^	46	0.29	23	0.36
All MZ	86	0.58	42	0.67
All DZ	86	0.29	42	0.35
MZ−2DZ	86	0.00	42	−0.04

These show the correlations (*r*) of phenotypes of twins, averaged over ranges of traits estimated in large data sets

a Data from published papers by N.G. Martin and colleagues of the Queensland Institute of Medical Research, Brisbane (www.genepi.edu.au)

b Opposite sex

## Model

### Gene Frequency Distributions

In view of the apparent conflict between the observations of high proportions of additive genetic variance (often half or more of the phenotypic variance, and even more of the total genetic variance) and the recent reports of epistasis at quantitative trait loci (QTL) [8], we consider explanations beyond that of simple sampling errors and bias of estimates. We focus particularly on the role that the distribution of gene frequencies may play in the relation between the genetic model and the observed genetic variance components.

Genetic variance components depend on the mean value of each genotype and the allele frequencies at the genes affecting the trait [3],[4],[17]. Unfortunately the allele frequencies at most genes affecting complex traits are not known, but the distribution of allele frequencies can be predicted under a range of assumptions. This distribution depends on the magnitude of the evolutionary forces that create and maintain variance, including mutation, selection, drift and migration. As the effects on fitness of genes at many of the loci influencing most quantitative traits are likely to be small, we can invoke theory for neutral alleles to serve as a reference point. An important such reference is the frequency distribution under a balance between mutation and random genetic drift due to finite population size in the absence of selection. If mutations are rare, the distribution of the frequency (*p*) of the mutant allele is f(*p*)∝1/*p*, i.e. approximately L-shaped [2],[35],[36], with the high frequency at the tail being due to mutations arising recently. The allele which increases the value of the trait may be the mutant or ancestral allele, so its frequency has a U-shaped distribution f(*p*)∝1/*p*+1/(1−*p*) = 1/[*p*(1−*p*)]. As we shall use it often, we define the ‘U’ distribution explicitly by this formula. For loci at which the mutants are generally deleterious, the frequency distribution will tend to be more concentrated near *p* = 0 or 1 than for this neutral reference point. As another simple reference point we use the uniform distribution, f(*p*)∝1, 1/(2*N*) ≤ *p* ≤ 1−1/(2*N*), with *N* the population size. This approximates the steady state distribution of a neutral mutant gene which has been segregating for a very long time [2], and also has much more density at intermediate gene frequencies than the ‘U’ distribution. Our third reference point is at *p* = 0.5, as in populations derived from inbred crosses, and is the extreme case of central tendency of gene frequency.

These analyses assume a gene frequency distribution which is relevant to no selection. For a more limited range of examples we consider the impact of selection on the partition of variance. We consider a limited range of genetic models, some simple classical ones and others based on published models of metabolic pathways or results of QTL mapping experiments.

*Uniform*: f(*p*) = 1, assuming *N* is sufficiently large that the discreteness of the distribution and any non-uniformity as *p* approaches 1 or 0 can be ignored, i.e. integrated over 0 to 1. This and the ‘U’ gene frequency distributions are, for simplicity, assumed to be continuous.

*Neutral mutation model (‘U’)*: f(*p*)∝1/[*p*(1−*p*)]. To standardise the distribution, with population size *N* assumed to be large, note that
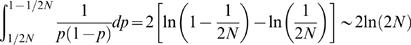



Thus 
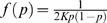
, where *K*∼ln(2*N*).

Genetic variance components are obtained by integration of expressions for the variance as a function of *p* for a specific model of the gene frequency distribution. For multiple locus models the distribution of all loci is assumed to be identical and there is no linkage disequilibrium. We focus on the contribution of additive genetic variance (*V*_A_) to genotypic variance (*V*_G_).

### Genotypic Values

#### Single Locus with Arbitrary Dominance

Consider a single biallelic locus with genotypic values for CC, Cc and cc of +*a*, *d* and −*a*, respectively (notation of [3]). Then, from [3]

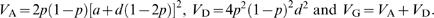



For the *uniform* distribution of *p*

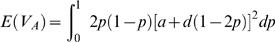



Hence E(*V*_A_) = *a* ^2^/3 +*d* ^2^/15 and E(*V*_D_) = 2*d* ^2^/15, giving E(V_A_)/E(V_G_) = 1−2*d* ^2^/(5*a* ^2^+3*d* ^2^).

For the *‘U’-distribution*, assuming *N* is large, and ignoring terms of O(1/*N*), the integrals simplify to E(*V*_A_)∼(*a* ^2^+*d* ^2^/3)/*K*, E(*V*_D_)∼*d* ^2^/(3*K*) and E(*V*_A_)/E(*V*_G_) = 1−*d* ^2^/(3*a* ^2^+2*d* ^2^).

#### Additive × Additive Model without Dominance or Interactions Including Dominance

A simple additive × additive epistatic model has these genotypic values:
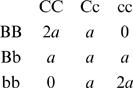



Assuming the frequency of B is *p* and of C *is q*, with linkage equilibrium:

Mean = *M* = 2*a*[*pq*+(1−*p*)(1−*q*)]

The average effect of substitution of allele B is given by [37]

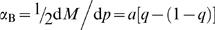
and hence

*V*_A_ = 2*a* ^2^[*p*(1−*p*)(1−2*q*)^2^+*q*(1−*q*)(1−2*p*)^2^] = *a* ^2^(*H_p_*+*H_q_*−4*H_p_H_q_*), where *H* is heterozygosity

The AA epistatic effect is given by (*αα*)_BC_ = ¼ d^2^ *M*/d*p*d*q* = *a*.

Hence *V*_AA_ = 4*a* ^2^ *p*(1−*p*)*q*(1−*q*)*a* ^2^ = *a* ^2^ *H_p_H_q_* and *V_G_* = *a* ^2^(*H_p_*+*H_q_*−3*H_p_H_q_*),

*Uniform*: simple integration gives E(*V*_A_) = 2*a* ^2^/9, E(*V*_AA_) = *a* ^2^/9, E(*V*_G_) = *a* ^2^/3

*‘U’*: 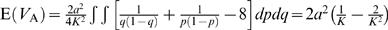


Similarly E(*V*_AA_) = *a* ^2^/(4*K*). Hence E(*V*_A_)/E(*V*_G_) = (2−4/*K*)/(2−3/*K*) = 1−1/(2*K*−3), which → 1 for large *K*. The residue, if any, is *V*_AA_.

#### Duplicate Factor Model

A simple epistatic model involving all epistatic components for two loci is the following:
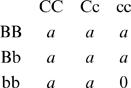



For an arbitrary number (*L*) of loci (*i*), the genotypic value is *a* except for the multiple ‘recessive’ homozygote, and for one locus it is complete dominance.
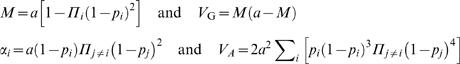



For *p_i_* = 0.5 at all loci: *V*_G_ = *a* ^2^[(½)^2*L*^−(¼)^2*L*^], *V*_A_ = *a* ^2^ *L*(½)^4*L*−1^ and *V*_A_/*V*_G_ = 2*L*/(2^2*L*^−1). For two loci, *V*_A_/*V*_G_ = 4/15.

*Uniform:* 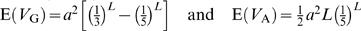


For two loci, E(*V*_A_)/E(*V*_G_) = 9/16 and declines to 0 as *L* increases.

*‘U’:* 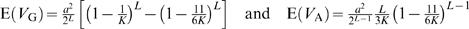


For two loci 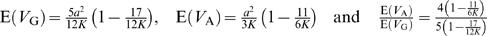


For large *N*, with two loci E(*V*_A_) /E(*V*_G_) → 4/5 and for very many loci E(*V*_A_) /E(*V*_G_) → 0

#### Complementary Model

Another simple epistatic model involving all components is the following:
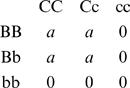
which can also be defined for multiple loci. For two loci, for example, using similar methods it can be shown that: for *p_i_* = 0.5, *V*_A_/*V*_G_ = 0.56; with the uniform distribution, E(*V*_A_)/E(*V*_G_) = 2/3; and with the ‘U’ distribution 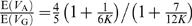.

#### Analyses of General Models

For two-locus models in which the genotypic values were not functions of simple parameters, the genotypic values were entered as data, and *V*_A_ and *V*_G_ calculated as functions of the gene frequencies *p* and *q*. Bivariate numerical integration was undertaken using Simpson's rule by computing e.g. *V*_A_(*p*,*q*)f(*p*,*q*) over an (*m*+1) × (*m*+1) grid of equally spaced *p* and *q* values, taking *m* = 2^10^ or higher power of 2 as necessary for adequate convergence. Results were computed for some models of metabolic pathways [38],[39] and for some published models obtained from QTL analysis [8].

## Results/Discussion

### Single Locus Model

Many general points are illustrated by two simple examples, the single locus model with dominance and the two locus model with AA interaction, so we consider these in more detail. For the single locus model with genotypic values for CC, Cc and cc of +*a*, *d* and −*a*, respectively, *V*_A_ = 2*p*(1−*p*)[*a*+*d*(1−2*p*)]^2^ and *V*_D_ = 4*p* ^2^(1−*p*)^2^ *d* ^2^. For *d* = *a*, i.e. complete dominance of C, *V*_A_ = 8*p*(1−*p*)^3^ *a* ^2^ and *V*_D_ = 4*p* ^2^(1−*p*)^2^ *a* ^2^ and thus: at *p* = 0.5, *V*_A_ = (2/3)*V*_G_; if the dominant allele is rare (i.e. *p* → 0), *V*_G_ → 8*p* and *V*_A_/*V*_G_ → 1, and if it is common, *V*_G_ → 4*p* ^2^ and *V*_A_/*V*_G_ → 0. Note, however, that *V*_G_ and *V*_A_ are much higher when the dominant allele is at low frequency, e.g. 0.1, than are *V*_G_ and *V*_D_ when the recessive is at low frequency, e.g. *p* = 0.9. Even for an overdominant locus (*a* = 0), all genetic variance becomes additive at extreme gene frequencies. Considering now expectations (E) over the frequency distributions, let *η* ^2^ = E(*V*_A_)/E(*V*_G_), an equivalent to narrow sense heritability if *V*_E_ = 0. For the ‘U’ distribution, *η* ^2^ = 1−*d* ^2^/(3*a* ^2^+2*d* ^2^) and for the uniform distribution, *η* ^2^ = 1−2*d* ^2^/(5*a* ^2^+3*d* ^2^). Hence, for a completely dominant locus, *η* ^2^ = 0.8 and *η* ^2^ = 0.75 respectively; whereas *V*_A_/*V*_G_ = 0.67 for *p* = 0.5. In summary, the fraction of the genetic variance that is additive genetic decreases as the proportion of genes at extreme frequencies decreases (Table 2).

**Table 2:** Summary of expected proportion of *V*_G_ that is *V*_A_ for different models^a^.

Genetic model	Distribution of allele frequencies			
	*p* = ½	Uniform	‘U’ (*N* = 100)^b^	‘U’ (*N* = 1000)
Dominance without epistasis *d* = ½*a*	0.89	0.91	0.93	0.93
Dominance without epistasis *d* = *a*	0.67	0.75	0.80	0.80
Dominance without epistasis *a* = 0	0.00	0.33	0.50	0.50
A × A without dominance	0.00	0.67	0.87	0.92
Duplicate factor 2 loci	0.27	0.56	0.71	0.75
Duplicate factor 100 loci	0.00	0.00	0.00	0.00
Complementary 2 loci	0.57	0.67	0.74	0.76

a Models defined in Methods section

b Population size

### Two Locus Additive × Additive Model

The genotypic values (see Theory section) for the simple AA model for double homozygotes BBCC and bbcc are +2*a* and for bbCC and BBcc are 0, and all single or double heterozygotes are intermediate (+*a*). With linkage equilibrium, *V*_A_/*V*_G_ = 1−*H_p_H_q_*/[*H_p_*+*H_q_*−3*H_p_H_q_*], where the heterozygosities are *H_p_* and *H_q_* at loci B and C. Thus *V*_A_/*V*_G_ → 1 if *either* locus is at extreme frequency (i.e. *p* or *q* → 0 or 1), and equals 0 when *p* = *q* = 0.5. If *p* = *q*, for gene frequencies 0.1, 0.2, 0.3 and 0.4, *V*_A_/*V*_G_ = 0.88, 0.69, 0.43 and 0.14. For the uniform distribution *η* ^2^ = 2/3, and for the ‘U’ distribution, the variances are a function of the population size, because more extreme frequencies are possible at larger population sizes. Thus *η* ^2^ = (2−4/*K*)/(2−3/*K*), where *K* = ln(2*N*), so *η* ^2^ → 1 for large *K*. Any residue is *V*_AA_.

These two examples, the single locus and A × A model, illustrate what turns out to be the fundamental point in considering the impact of the gene frequency distribution. When an allele (say C) is rare, so most individuals have genotype Cc or cc, the allelic substitution or average effect of C vs. c accounts for essentially all the differences found in genotypic values; or in other words the linear regression of genotypic value on number of C genes accounts for the genotypic differences (see [3], p 117). Hence almost all *V*_G_ is accounted for by *V*_A_.

### Other Epistatic Models

With the ‘U’ distribution, most genes have one rare allele and so most variance is additive. Further examples (Table 2) illustrate this point, including the duplicate factor and complementary models where there is substantial dominance and epistasis. These models show mostly *V*_A_ for the ‘U’ distribution for a few loci but the proportion of the variance which is additive genetic declines as the number increases. With many loci, however, such extreme models do not explain the covariance of sibs (i.e. any heritability) or the approximate linearity of inbreeding depression with inbreeding coefficient, *F*, found in experiments [3],[4],[40],[41],[42], or the linearity in response to artificial selection [43].

We also analysed a well-studied systems biology model of flux in metabolic pathways [38],[39],[44] and found again that the expected proportion of *V*_G_ that is accounted for by *V*_A_ is large (Table 3).

**Table 3:** Examples of expected proportion of *V*_G_ that is *V_A_* in models of flux in linear metabolic pathways with a model flux *J*∝[Σ*_i_*(1/*E_i_*)]^−1^ for a system with 10 loci in which 8 are invariant wild type and two (B, C) are mutants.

Activities		Flux relative to wildtype, *J*_BBCC_ = 1				E(*V*_A_)/E(*V*_G_)			
*E*_bb_	*E_cc_*	*J*_BbCc_	*J*_bbCC_	*J*_BBcc_	*J*_bbcc_	Distribution of allele frequencies			
						0.5	Uni^a^	U100^b^	U1000^c^
1	0.1	0.92	1	0.53	0.53	0.81	0.86	0.88	0.88
0.5	0.1	0.90	0.91	0.53	0.50	0.81	0.85	0.88	0.88
0.1	0.1	0.86	0.53	0.53	0.36	0.77	0.82	0.86	0.87
0.1	0.01	0.85	0.53	0.09	0.09	0.72	0.79	0.83	0.84

Enzyme activities are *E_i_* = 1 for loci 3 to 8, *E*_BB_ = *E*_CC_ = 1, values of *E*_bb_ and *E*_cc_ are listed, and heterozygotes are intermediate, e.g. *E*_Cc_ = ½(1+*E*_cc_), assuming gene frequency distributions as in Table 2. Flux modelled as [39].

a Uniform

b U-shaped with population size of 100

c U-shaped with population size of 1000

### Examples of Models from Highly Epistatic Published QTL Analyses

A number of QTL analyses using crosses between populations (some inbred, some selected) have been published in which particular pairs (or more) of loci have been identified to have substantial epistatic effects [8]. We consider examples of the more extreme cases of epistasis found, obtaining variance components by numerical integration. Results are shown in Table 4, for examples from [8] deliberately chosen as extreme. Even so, the proportion of the genetic variance that is additive is high with the ‘U’ distribution, except in the dominance × dominance example. Further, as these examples were selected by Carlborg and Haley and us as cases of extreme epistasis, it is not unreasonable to assume that the real epistatic effects are smaller than their estimates.

**Table 4:** Examples of expected proportion of *V*_G_ that is *V*_A_ in highly epistatic published QTL analyses assuming gene frequency distributions as in Table 2.

Model^a^	Genotypic values									E(*V*_A_)/E(*V*_G_)			
	BBCC	BbCC	bbCC	BBCc	BbCc	bbCc	BBcc	Bbcc	bbcc	Distribution of allele frequencies			
										0.5	Uni^b^	U100^c^	U1000^d^
DomEp	4	10	15	11	8	7	10	8	7	0.05	0.52	0.73	0.78
Co-ad	39.0	38.7	35.7	37.6	38.9	37.7	36.8	39.6	40.4	0.11	0.62	0.81	0.85
D × D	4	13	6	13	7	11	5	13	6	0.00	0.15	0.37	0.42

a Values obtained from tables or by interpolation from Box 1c–e of Carlborg and Haley [8]: key to their nomenclature: DomEp: Dominant epistasis (complex); Co-ad: Co-adaptive epistasis; D × D: dominance × dominance epistasis.

b Uniform.

c U-shaped with population size of 100.

d U-shaped with population size of 1000.

### Relaxation of Assumptions

#### Expectation of a Ratio of Variance Components

The formulae we have given have been for the quantities E(*V*_A_), E(*V*_G_) and the ratio E(*V*_A_)/E(*V*_G_). The quantity actually observed is *V*_A_/*V*_G_ = Σ*_i_V*_A*i*_/Σ*V*_G*i*_ where the expression denotes the sums over loci (*i*) of the additive and total genetic variance contributed by each in the absence of epistasis or linkage disequilibrium, or in the presence of these, sums over relevant sets of loci. As, for any locus, or for their sum, in general E(*V*_A_/*V*_G_) ≠ E(*V*_A_)/E(*V*_G_), we need to consider the relevance of the quantities calculated. Whilst it would be possible to obtain approximations using statistical differentiation [4], formulae are complicated and invoke an assumption of small coefficients of variation of the quantities which does not always hold. Hence we used Monte Carlo simulation and some examples are given in Table 5. It is seen that, except with very few loci, the bias is not great in using the ratio of expectations. In real situations where many loci of differing effects and frequencies are likely to be involved, the bias is likely to be trivial unless a single locus contributes almost all the variance.

**Table 5:** Bias in use of E(*V*_A_)/E(*V*_G_) rather than E(*V*_A_/*V*_G_) for some models in Table 2 as a function of Numbers of Loci.

	Uniform distribution				
	E(*V*_A_)/E(*V*_G_)	E(*V*_A_/*V*_G_) from simulation			
Loci^a^		64	16	4	1
*a* = 1, *d* = 1	0.750	0.749	0.747	0.734	0.609
*a* = 0, *d* = 1	0.333	0.335	0.337	0.348	0.430
A × A	0.667	0.667	0.666	0.660	0.646
Dupl. factor	0.562	0.559	0.549	b	b
	‘U’ distribution with *N* = 1000				
	E(*V*_A_)/E(*V*_G_)	E(*V*_A_/*V*_G_) from simulation			
Loci*		64	16	4	1
*a* = 1, *d* = 1	0.800	0.798	0.796	0.773	0.561
*a* = 0, *d* = 1	0.500	0.502	0.516	0.585	0.800
A × A	0.918	0.918	0.919	0.925	0.945
Dupl. factor	0.746	0.743	0.733	b	b

a Number of loci for non-epistatic cases (complete dominance *a* = 1, *d* = 1, and overdominance *a* = 0, *d* = 1), numbers of pairs of loci for two-locus epistatic models (A × A and duplicate factor.

b Not computed as *V*_G_ = 0 in some replicates.

#### Influence of Linkage Disequilibrium (LD)

In this analysis we have assumed there is Hardy-Weinberg equilibrium (HWE) and linkage equilibrium among the loci. As departures from HWE are transient with random mating, they can be ignored, but LD can persist, and hence the estimated effects at locus C depend on those fitted at B and vice versa. The effect of LD is to reduce the number of haplotypes that segregate in the population so what would be epistatic variance becomes additive or dominance variance. For example, consider the A × A model and complete LD, i.e. equal frequencies at B and C loci and both loci segregating but with only two haplotypes present. Then only Bc and bC haplotypes are present, and genotypic values are 0 for homozygous classes and *a* for heterozygotes (‘pure’ overdominance), or only BC and bc haplotypes, with genotypic values 2*a* for homozygotes and *a* for heterozygotes (‘pure’ underdominance). In either case variances are the same as for the dominance case with *a* = 0. Thus LD would lead to attribution of real epistatic variance to additive or dominance variance, and would exacerbate the results obtained from discussions of gene frequency distribution.

#### Consequences of Multiple Alleles

In these models we have considered solely biallelic loci, appropriate for low mutation rates. Multiallelic loci, in terms of their effects on the trait, can arise from mutations at different structural or control sites. Predictions are complicated by the need to consider *k*(*k*−1)/2 genotypic values at a *k* allelic locus, and many further epistatic terms, so we consider two extreme cases. If the alleles all have similar effects, for example due to a knock-out, the effective mutation rate is increased, but it would require very many such sites for the distribution of frequencies of the trait alleles to differ greatly from proportionality to 1/[*p*(1−*p*)]. Such segregation of multiple alleles will be more common in large populations, where in any case the frequency distribution is most extreme, and so the impact is unlikely to be large. A second case is where all alleles have different effects and dominance interactions. Any allelic substitution then produces a change in the mean and so additive variance is present and for example, contributes more *V*_A_ than does the overdominance model at *p* = 0.5.

#### Alternative Models

The analysis we have given for estimating effects of dominance and epistasis is for the classical method using simple averages over genotypes weighted by their frequencies, which are the least squares estimates in the balanced case and the basis for the analysis of variance [14],[15],[16]. There are alternative parameterisations aimed at exemplifying more clearly the nature of the interactions, including that of ‘physiological epistasis’ [45]. Whilst such alternatives may be of use in the analysis and interpretation of gene or QTL mapping experiments where individual genotypes can be identified or predicted from linked markers, such alternative parameterizations are not feasible in analysis of populations using data solely on the quantitative traits, from which the estimates of genetic variance components and heritability are obtained. Further, as has been pointed out [46], although the estimated effects may differ, the variances explained by different models are generally the same in segregating populations.

#### Effects of Selection on Gene Frequency Distributions and Partition of Variance

The ‘U’ and indeed uniform gene frequency distributions are limiting cases applying in the absence of selection on loci affecting the quantitative trait. The results for a wide range of models can be summarised as follows: gene frequencies that cause *V*_A_/*V*_G_ to be small also cause *V*_G_ to be small. Consequently, when *V*_A_ and *V*_G_ are summed over a full range of frequencies, *V*_A_/*V*_G_ is large. This conclusion is dependent on the distribution of gene frequencies being symmetrical, so that cases with large *V*_G_ and large *V*_A_/*V*_G_ are as common as cases with small *V*_G_ and small *V*_A_/*V*_G_. The impact of selection will depend on how it acts on the trait or traits analysed and also on other aspects of fitness, so we need to consider whether the findings are robust to selection.

Stabilising selection on the trait, such that individuals with phenotype closest to an optimum are most fit, leads to maintenance of the population mean at or close to the optimum, so that mutants are at a disadvantage if they increase or decrease trait values. Consequently the gene frequency distribution is still broadly U-shaped, but with much more concentration near 0 or 1 [47]. Hence such selection is likely to increase proportions of additive variance. This conclusion would be wrong if there was widespread overdominance at the level of individual genes because this would push gene frequencies to intermediate values. However, the observed inbreeding depression is incompatible with widespread overdominance [48].

Under the neutral mutation or stabilising selection models where gene frequency distributions have extreme U shape, subsequent directional selection will lead to either rapid fixation or increase to intermediate frequency of genes affecting the trait. Even if the distribution of allele frequencies is initially symmetric, a net increase in variance over generations might thus be expected [49] (Chapter 6). Accelerated responses to artificial selection have not been seen, however, in lines founded from natural populations [50]. Calculations show that if genes are analysed independently such an increase in variance with artificial selection can in theory occur following the neutral model only if most gene effects are large (unpublished) or with more extreme frequency distributions following stabilising selection [51]. These ignore the build up of negative gametic disequilibrium through the Bulmer effect [52], however, whereas in simulated multi-locus models of Drosophila no increase in variance was found [51]. Linkage effects would be weaker in species with more chromosomes, but selection lines in these have typically not been founded directly from natural populations.

Other types of selection do lead to an asymmetrical distribution of allele frequencies because the unfavourable allele will typically be at a low frequency. We have considered the case of genes whose effect on both the trait measured and on fitness shows complete dominance. Thus recessive and dominant favourable and unfavourable mutants were considered, and their expected contribution to variance computed during their lifetime to fixation or loss, using transition matrix methods. Results are given in Table 6 for population size (*N*) 100 and selective values (*s*) of the homozygote of 0.05 (*Ns* = 5), but the qualitative result is not affected by using weaker or stronger selection. Deleterious, recessive mutations show the lowest *V*_A_/*V*_G_ but even here it is 0.44 and these cases also show the lowest total variance. Consequently, in a trait affected by a mix of genes with varying types of gene action, *V*_A_/*V*_G_ is likely to be well above 0.5.

**Table 6:** Expected variance contributed by mutant genes before fixation for population size 100, specified dominance on the quantitative trait (*a* vs *d*) and selective (dis)advantage (*s* in heterozygote and homozygote)^a^.

Model	*s*(het)	*s*(hom)	*a*	*d*	E(*V*_G_)	E(*V*_A_)/E(*V*_G_)
Neutral dominant	0	0	1	1	0.388	0.86
Neutral recessive	0	0	1	−1	0.166	0.66
Neutral random^b^	0	0	1	1 or −1	0.277	0.80
Deleterious dominant	−0.05	−0.05	1	1	0.145	0.97
Deleterious recessive	0	−0.05	1	−1	0.052	0.44
Advantageous dominant	0.05	0.05	1	1	0.375	0.74
Advantageous recessive	0	0.05	1	−1	0.151	0.71

a e.g., if the mutant gene is completely recessive for the trait and for fitness, *d* = −*a* and *s*(hom) = 0.

b Equally likely to be completely dominant or recessive mutants, hence values as in Table 2.

Thus if the highest and lowest genotypic values correspond to multiple homozygous classes, it is clear that a high proportion of the variance is expected to be additive genetic even with selection. The potential exceptions occur when there is a maximum at intermediate frequencies, such as with an overdominant locus or some of the cases shown in Table 4. Nevertheless, few confirmed cases of clear overdominance/heterozygote superiority have been found (other than sickle cell anaemia) and the patterns in Table 4 are somewhat erratic.

#### Effect of Population Size and Bottlenecks

The theoretical analysis has been undertaken for large populations but much of the experimental data comes from livestock, laboratory animals and humans, all of which have experienced bottlenecks of reduced effective population size. As has been much explored, bottlenecks of population size are likely to change the proportion of variation that is additive, and for example to increase levels of *V*_A_ for recessives at low frequency [53] and to ‘convert’ epistatic into additive variation [54],[55],[56],[57],[58], thereby increasing the ratio *V*_A_/*V*_G_. For example, for the additive × additive two locus model, the ratio of variances at inbreeding level *F* in terms of values at *F* = 0 is *V*_A_(*F*)/*V*_G_(*F*) = (*V*_A_+4*FV*_AA_)/(*V*_A_+*V*_AA_+3*FV*_AA_) for any gene frequency (using results of [54], but for loci with dominance or dominance interactions, *V*_A_(*F*)/*V*_G_(*F*) depends on gene frequency. This occurs because the bottleneck leads to the dispersal of gene frequencies and the reduction in mean heterozygosity, so for the AA model, if frequencies are initially intermediate (e.g. 0.5) there is a substantial increase in *V*_A_/*V*_G_, whereas if frequencies initially follow the ‘U’ distribution, there is little *V*_AA_ initially, total variance falls and the level of dispersion and *V*_A_/*V*_G_ do not increase appreciably. Indeed, for a population that starts with the gene frequency distribution U-shaped, the loss of heterozygosity is due to fixation. Among the genes that remain segregating the distribution of gene frequencies flattens considerably, and in the absence of new mutation approaches the uniform distribution which has a lower ratio of *V*_A_/*V*_G_ than the ‘U’ distribution. However, despite this, *V*_AA_ declines faster than *V*_A_ because, as loci become fixed, the number of pairs of segregating loci declines faster than the number of segregating loci. Thus it is not obvious what effect the bottlenecks in livestock, laboratory or human populations have had on the ratio *V*_A_/*V*_G_. We suspect it has not been large because, if a large reduction in heterozygosity had occurred, these populations would show low genetic variance and there is no indication that this is the case. In any case, the results show that the conclusion that most genetic variance is additive is fairly robust to assumptions about the distribution of gene frequencies, for instance the ‘U’ and uniform distributions both lead to qualitatively the same conclusion.

### Evidence for the Effect of Gene Frequency on Variance Components

A test of the hypothesis that the lack of non-additive variance observed in populations of humans or animals is because gene frequencies near 0.5 are much less common than those more extreme, not because non-additive effects are absent, is to compare variance components among populations with different gene frequency profiles. For crops such as maize and for laboratory animals, estimates can be got both from outbreds and from populations with gene frequencies of one-half derived from crosses of inbred lines. There are a limited number of possible contrasts and linkage confounds comparisons of variation in F_2_ and later *inter se* generations, however, so it is difficult to partition variation between single locus and epistatic components (e.g. [17] ch. 7).

The most extensive data are on yield traits in maize. The magnitudes of heritability and of dominance relative to additive variance estimated for different kinds of populations in a substantial number of studies (including 24 on F_2_ and 27 on open-pollinated, i.e. outbreds) have been summarised [59]. Average estimates of *h* ^2^ were 0.19 for open-pollinated populations, 0.23 for synthetics from recombination of many lines, 0.24 for F_2_ populations, 0.13 for variety crosses and 0.14 for composites. Estimates of *V*_A_/*V*_G_ (from tabulated values of *V*_D_/*V*_A_ [59]) were 0.57, 0.55, 0.50, 0.42 and 0.43, respectively, which are inconclusive but indicate relatively more dominance variance at frequencies of 0.5. Analyses of the magnitude of epistasis at the level of effects, rather than variance, do not provide consistent patterns. For example, in two recent analyses of substantial data sets of F_2_ populations of maize, one found substantial epistasis [60] and the other almost none [61]. In an analysis of a range of traits in recombinant inbred lines, F_2_ and triple test crosses [62] in *Arabidopsis thaliana*, there was substantial additive genetic and dominance variance for all traits, with most estimates of *V*_D_/*V*_A_ in the range 0.3 to 0.5, essentially no significant additive × additive epistatic effects, but several cases of epistasis involving dominance [63].

Although there does appear to be more dominance variance in populations with gene frequencies of one-half than with dispersed frequencies, from these results we cannot reject or accept the hypothesis that there is relatively much more epistatic variance in such populations. One explanation is indeed that there is not a vast amount of epistatic variance in populations at whatever frequency, although another is that maize has unusually small amounts of epistasis. Many additive QTL were identified in an analysis of a line derived from the F_2_ of highly divergent high and low oil content lines from the long term Illinois maize selection experiment, but with almost no evidence of epistasis or indeed dominance effects [64]. In contrast, an F_2_ of divergent lines of long-term selected poultry and an F_2_ from inbred lines of mice showed evidence of highly epistatic QTL effects for body weight [65],[66]. We do not claim to understand these different results, but as has been pointed out [67],[68], QTL with significant epistatic interaction effects might not represent the majority of QTL with small effects contributing to gene networks.

### Conclusions and Consequences

We have summarised empirical evidence for the existence of non-additive genetic variation across a range of species, including that presented here from twin data in humans, and shown that most genetic variance appears to be additive genetic. There are two primary explanations, first that there is indeed little real dominant or epistatic gene action, or second that it is mainly because allele frequencies are distributed towards extreme values, as for example in the neutral mutation model. Complete or partial dominance of genes is common, at least for those of large effect; and epistatic gene action has been reported in some QTL experiments [8],[69]. Detailed analyses in *Drosophila melanogaster*, using molecular and genetic tools available for it, identify substantial amounts of epistasis, including behavioural traits [70] and abdominal bristle number [71], yet most genetic variation in segregating populations for bristle number appears to be additive (as noted above). But many QTL studies of epistatic gene action suffer from a high degree of multiple testing, increasingly so the more loci and orders of interaction are included, such that they may be exaggerating the amount of epistasis reported. On the assumption that many of the effects are indeed real, we have turned our attention to the second explanation.

The theoretical models we have investigated predict high proportions of additive genetic variance even in the presence of non-additive gene action, basically because most alleles are likely to be at extreme frequencies. If the spectrum of allele frequencies is independent of which are the dominant or epistatic alleles, *V*_A_/*V*_G_ is large for almost any pattern of dominance and epistasis because *V*_A_/*V*_G_ is low only at allele frequencies where *V*_G_ is low, and so contributes little to the total *V_G_*. The distribution of allele frequencies is expected to be independent of which are the dominant or epistatic alleles for neutral polymorphisms; but under natural selection the favourable allele is expected to be common and lead to high or low *V*_A_/*V*_G_ depending on whether it is dominant (low *V*_A_) or recessive (high *V*_A_). The equivalent case for epistasis is that all genotype combinations except one is favourable (low *V*_A_) vs. only one genotype combination is favourable (high *V*_A_).

If genetic variation in traits associated with fitness is due almost entirely to low frequency, deleterious recessive genes which are unresponsive to natural selection, these traits would show low *V*_A_/*V*_G_. However, neither the empirical evidence nor the theory supports this expectation. There seems to be substantial additive genetic variance for fitness associated traits [21] and fitness itself [30],[31],[72]. Although heritabilities for such traits may be low, they show high additive genetic coefficient of variation (evolvability) [29], and the correlation of repeat records is typically little higher than the heritability (e.g., litter size in pigs), indicating that *V*_A_/*V*_G_ is one-half or more. In agreement with this, when the life history of deleterious, recessive mutants was modelled, *V*_A_/*V*_G_ was found to be 0.44 (Table 6), basically because rare recessives contribute so little variance, albeit most is *V*_D_, in non-inbred populations.

We believe we have a plausible gene frequency model to explain the minimal amounts of non-additive genetic and particularly epistatic variance. What consequences do our findings have? For animal and plant breeding, maintaining emphasis on utilising additive variation by straightforward selection remains the best strategy. For gene mapping, our results imply that *V*_A_ is important so we should be able to detect and identify alleles with a significant gene substitution effect within a population. Such variants have been reported from genome-wide association studies in human population [9],[10],[11],[12],[13]. Although there may well be large non-additive gene effects, the power to detect gene-gene interactions in outbred populations is a function of the proportion of variance they explain, so it will be difficult to detect such interactions unless the effects are large *and* the genes have intermediate frequency. Thus we expect that the success in replicating reported epistatic effects will be even lower than it is for additive or dominance effects, both because multi-locus interactions will be estimated less accurately than main effects and because they explain a lower proportion of the variance. Finally, if epistatic effects are real, gene substitution effects may vary widely between populations which differ in allele frequency, so that significant effects in one population may not replicate in others.
